# Supplementary material for: Organohydrogel-based transparent terahertz absorber via ionic conduction loss
Source: Nat Commun. 2024 Jan 2;15:38. doi: 10.1038/s41467-023-44344-2 (PMC10762151; doi:10.1038/s41467-023-44344-2)
Supplement: Supplementary file 1 — Supplementary Information [file 41467_2023_44344_MOESM1_ESM.pdf]

Supplementary Information

Organohydrogel-based transparent terahertz absorber via ionic conduction loss

This PDF file includes:

Supplementary Note 1. Terahertz dielectric parameter extraction

Supplementary Note 2. Finite element simulation of terahertz absorption

Supplementary Note 3. Calculation of conduction loss and polarization loss

Supplementary Figs. 1 to 26

Supplementary Table 1

Supplementary References

Supplementary Note 1. Terahertz dielectric parameter extraction

The terahertz (THz) dielectric/optical parameters are extracted from the transmitted THz signal of the sample ($\tilde{E}_{\mathrm{sam}}(\omega)$) and the reference ($\tilde{E}_{\mathrm{ref}}(\omega)$), which are Fourier transformed from the time-domain measurements. The optical parameters including refractive index $n(\omega)$ and extinction coefficient $k(\omega)$ are firstly deduced from the complex transfer function $\widetilde{H}(\omega)$ via:

$$\widetilde{H}(\omega) = \frac{\tilde{E}_{\mathrm{sam}}(\omega)}{\tilde{E}_{\mathrm{ref}}(\omega)} = \frac{4n(\omega)}{[1+n(\omega)]^2} \cdot \exp\left\{-k(\omega)\frac{\omega d}{c}\right\} \cdot \exp\left\{-i[n(\omega)-1]\frac{\omega d}{c}\right\} \quad (1)$$

Where d is the thickness of sample, ω is the angular frequency of THz wave, c is the vacuum speed of light. Based on the refractive index and extinction coefficient, the complex permittivity $\tilde{\varepsilon}(\omega)$ and the attenuation coefficient $\alpha(\omega)$ are calculated from the following equations:

$$\tilde{\varepsilon}(\omega) = \varepsilon'(\omega) + i\varepsilon''(\omega) = \big(n(\omega) + ik(\omega)\big)^2 \quad (2)$$

$$\alpha(\omega) = \frac{2\omega k(\omega)}{c} \quad (3)$$

The impedance matching value $Z_{\mathrm{c}}(\omega)$ is obtained by:

$$Z_{\mathrm{c}}(\omega) = \sqrt{\mu/\tilde{\varepsilon}(\omega)} \quad (4)$$

Where μ is the permeability, which equals 1 here.

Supplementary Note 2. Finite element simulation of terahertz absorption

The commercial software COMSOL Multiphysics 5.4 (COMSOL, Stockholm, Sweden) was used to simulate the THz absorption of the materials in alternating electromagnetic fields. A radio frequency (RF) physics field was applied in the finite element simulation process. A 0.5×1 cm rectangular sandwiched by two 0.1×1 cm rectangular with measured electromagnetic parameters and ionic conductivities were designed to represent the PDA@OHG. Perfectly matched conditions were imposed on the air domain to eliminate interference from the reflected waves. Two ports (port 1 and port 2) were established in the air domain to calculate the S-parameters. Port 1 was activated with 1 W m^{-1} input power in electric field mode. The COMSOL simulation was conducted in the steady analysis mode.

Supplementary Note 3. Calculation of conduction loss and polarization loss

According to the Debye theory, the complex permittivity is described by following equations:

$$\varepsilon' = \varepsilon_\infty + \frac{\varepsilon_s - \varepsilon_\infty}{1+\omega^2\tau^2} \tag{5}$$

$$\varepsilon'' = \frac{\varepsilon_s - \varepsilon_\infty}{1+\omega^2\tau^2}\omega\tau + \frac{\sigma}{\omega\varepsilon_0} \tag{6}$$

The imaginary part ε'' represents the dielectric loss, which can be divided into two parts: polarization loss (ε''_p) and conductive loss (ε''_c):

$$\varepsilon''_p = \frac{\varepsilon_s - \varepsilon_\infty}{1+\omega^2\tau^2}\omega\tau \tag{7}$$

$$\varepsilon''_c = \frac{\sigma}{\omega\varepsilon_0} \tag{8}$$

where ω is the angular frequency, ε_s is the stationary dielectricconstant, ε_∞ is the optical dielectric constant, σ is the electrical conductivity, τ is the relaxation time.

Non-linear least squares fitting was used to fit the parameters ε_s, ε_∞, σ, and τ, which are signed as a group *p*. The sum of squares is $S = \sum_{i=1}^{m} r_i^2$, where r_i is ε_{fit}-ε. Then our goal is minimizing the *S* by adjusting *p*. In order to fit these data as accurate as possible, our original data are divided into 40 parts and fitted separately. The mean number is the final result. Also, we use a Python package Scipy* to simplify our code. The detailed code is shown following.

```
import numpy as np
from scipy.optimize import least_squares
import xlrd
import matplotlib.pyplot as plt
import xlwt

def realimag(array):
return np.array([(x.real, -x.imag) for x in array])
```

```
def func(x,p):
s,u,sigma,t=p    #s:static dielectric constant;u:optical dielectric
constant;sigma:conductivity;t:relaxation time
    d = complex(0,1)
    o = 8.854187817*10**(-12)
return realimag(u+(s-u)/(1+np.dot(d,np.dot(x,t)))-
np.dot(d,np.divide(sigma,np.dot(x,o))))

def conduct_loss_result(x,p):
    s,u,sigma,t=p
    o=8.854187817*10**(-12)
return np.divide(sigma,np.dot(x,o))

def relax_loss_result(x,p):
    s,u,sigma,t=p
return np.dot(np.divide(s-u,(1+np.dot(x,t)**2)),np.dot(x,t))

def residuals(p,y,x):
return (realimag(np.array(y)) - func(x,p)).flatten()

p0 = [50,5,1,10**-11]
data=xlrd.open_workbook(r'original_data. xlsx')
table=data.sheets()[0]
fcost=0
fsig=0                          #fitting conductivity
ft=0                            #fitting relaxation time
fplsq=[]
fs=0                            #fitting static dielectric constant
fu=0                            #fitting optical dielectric constant
fconduct_loss=0                 #fitting conducting loss
```

```
frelax_loss=0                    #fitting polarization loss
for i in range(1,41):
    end=i*10
    start=end-10
    xdata=table.col_values(0)[start:end]       #experimental angular frequency
    ydata_1=table.col_values(2)[start:end]    #experimental imaginary permittivity
    ydata_2=table.col_values(1)[start:end]    #experimental real permittivity
    ydata=[]
    ydata1_mean=np.mean(ydata_1)
    ydata2_mean=np.mean(ydata_2)
    for i in range(10):
        ydata.append(complex(ydata_2[i],-ydata_1[i]))
    plsq = least_squares(residuals, p0, bounds=([0,0,0,0],[100,100,100,10**-
10]),args=(ydata, xdata),max_nfev=100000)
    fplsq.append(plsq)
    fs = plsq.x[0]
    fu = plsq.x[1]
    fsig = plsq.x[2]
    ft = plsq.x[3]
    fcost = plsq.cost
    fconduct_loss = np.mean(conduct_loss_result(xdata, plsq.x))
    frelax_loss = np.mean(relax_loss_result(xdata, plsq.x))
    fydata2 = fu + np.divide((fs - fu), (1 + (np.dot(xdata, ft) ** 2)))
    fydata1 = np.dot(np.divide((fs - fu), (1 + (np.dot(xdata, ft) ** 2))), np.dot(xdata, ft))
+ np.divide(fsig, np.dot(xdata, o))
    print(f'{frequency}, {fconduct_loss}, {frelax_loss}, {np.mean(fydata1)},
{ydata1_mean}, {fs}, {fu}, {fsig}, {ft}')
```

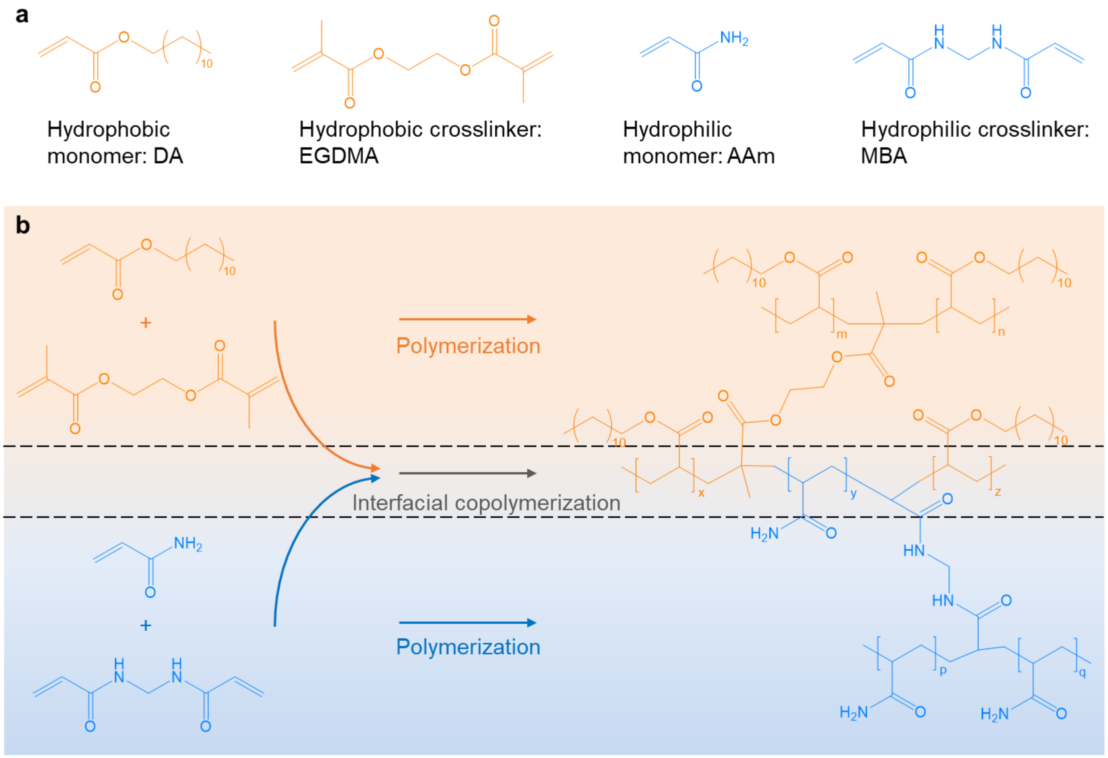

Supplementary Fig. 1 | Schematic diagram of the polymerization reaction of polydodecyl acrylate coated organohydrogel (PDA@OHG). a Structural formula of the chemical compositions required for the preparation of PDA@OHG. DA: dodecyl acrylate, EGDMA: ethylene glycol dimethacrylate, AAm: acrylamide, MBA: N,N′-methylenebis(acrylamide). **b** Diagram of copolymerization during the preparation of PDA@OHG.

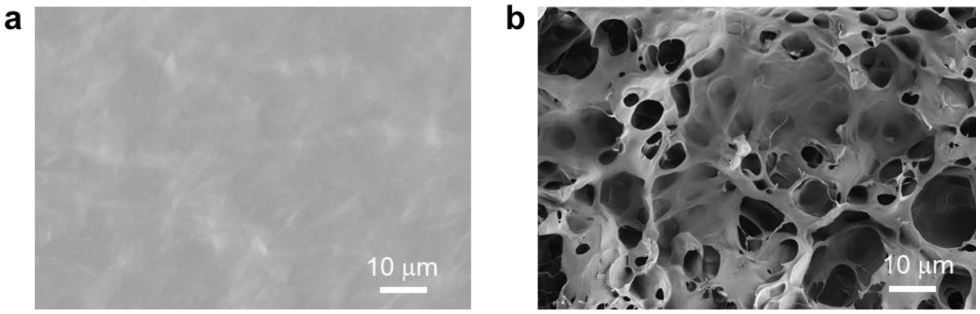

Supplementary Fig. 2 | Morphology of polydodecyl acrylate (PDA) and organohydrogel (OHG). a Scanning electron microscope (SEM) image of the PDA layer, showing a nonporous morphology. **b** SEM image of the freeze-dried OHG layer, showing a porous morphology.

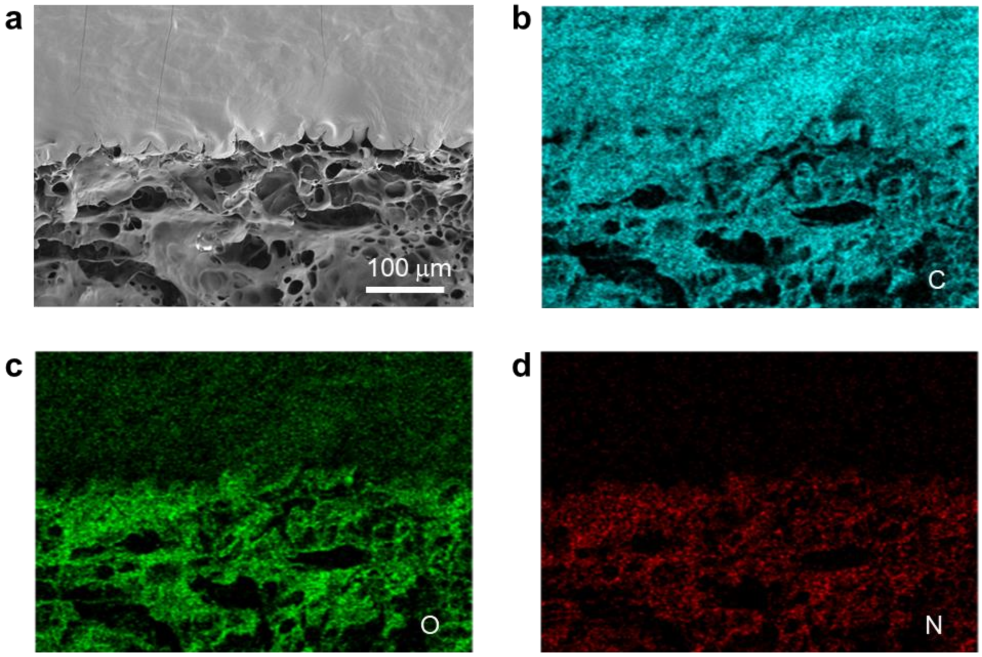

Supplementary Fig. 3 | The element distribution of polydodecyl acrylate coated organohydrogel (PDA@OHG). a Cross-section scanning electron microscope (SEM) image of the PDA@OHG. **b-d** Corresponding energy dispersive spectrometer (EDS) mapping of carbon (b), oxygen (c), and nitrogen element (d), representing the chemical components of the PDA@OHG.

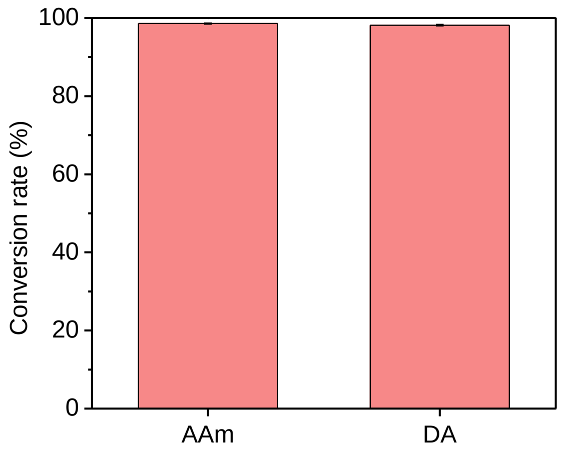

Supplementary Fig. 4 | Conversion rate of acrylamide (AAm) and dodecyl acrylate (DA). Each error bar represents the standard deviation from 5 measurements. Source data are provided as a Source Data file.

The conversion rate of the AAM (or DA) is obtained by measuring the weight ratio of the polymerized polyacrylamide (PAM) (or polydodecyl acrylate (PDA)) to the AAM (or DA) monomer. The PAM organohydrogel (OHG) and PDA samples were firstly prepared through the polymerization method. Note that the OHG (or PDA) samples were polymerized under a 100-μm-thick PDA (or 500-μm-thick OHG) layer to mimic the polymerization conditions of the polydodecyl acrylate coated organohydrogel (PDA@OHG) film. Then the as-prepared samples were dried in a vacuum drying oven of 60 °C for 24 h to the mass of m_1 (this drying process can be omitted for PDA samples). After that the OHG and PDA samples were respectively immersed in pure water and ethyl acetate for 24 h to remove the unreacted monomers, and then they were dried again in the vacuum drying oven of 60 °C for 24 h to the mass of m_2. The conversion rate of AAM (or DA) equals m_2/m_1.

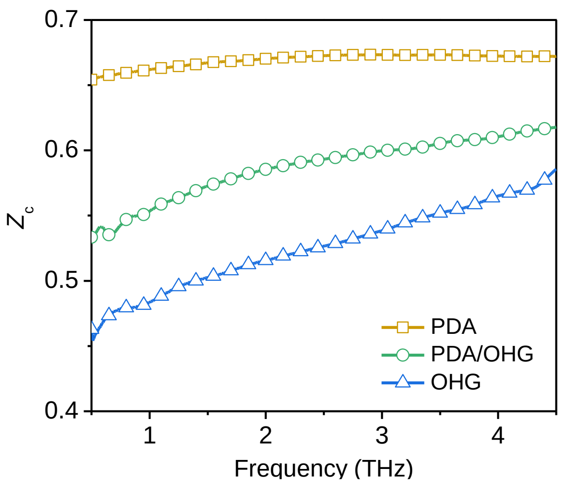

Supplementary Fig. 5 | Impedance matching values (Z_c) of 500-μm-thick polydodecyl acrylate (PDA), organohydrogel (OHG), and PDA/OHG mixture in 0.5-4.5 THz band. Source data are provided as a Source Data file.

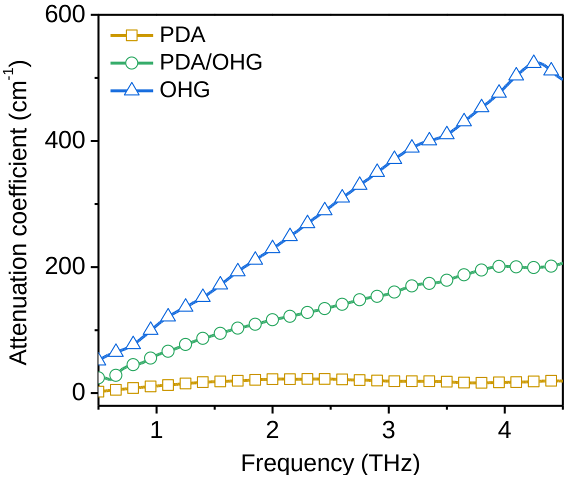

Supplementary Fig. 6 | Attenuation coefficients of polydodecyl acrylate (PDA), organohydrogel (OHG), and PDA/OHG mixture in 0.5-4.5 THz band. Source data are provided as a Source Data file.

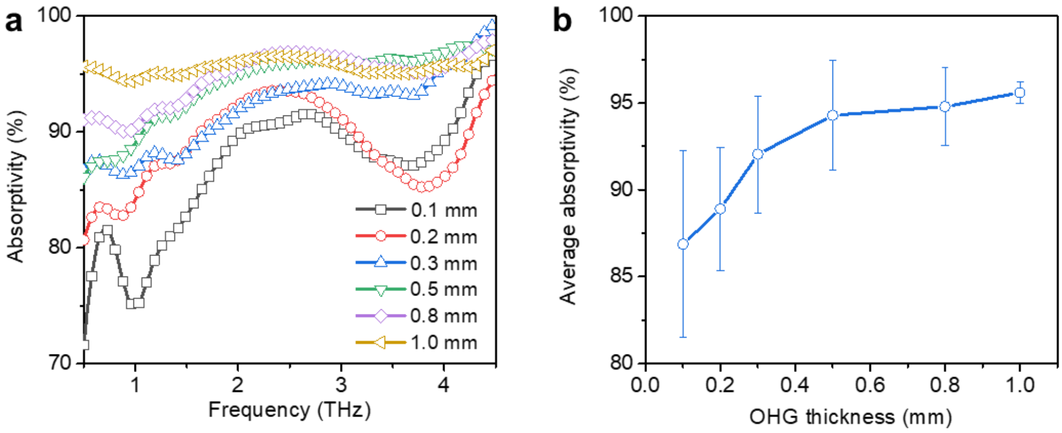

Supplementary Fig. 7 | Absorptivity of organohydrogel (OHG) with different thickness in 0.5-4.5 THz band. a Absorptivity curves of OHG with different thickness in the frequency range of 0.5-4.5 THz. **b** Average absorptivity of OHG as a function of thickness. Each error bar represents the standard deviation from 5 measurements. Source data are provided as a Source Data file.

The average absorptivity of OHG increases from 86.87% to 94.29% as the thickness increases from 0.1 mm to 0.5 mm, and then maintains at about 94% with the increasement of thickness from 0.5 mm to 1 mm. Considering the requirement of light and thin for terahertz absorbers, the optimal thickness of OHG layer is 0.5 mm.

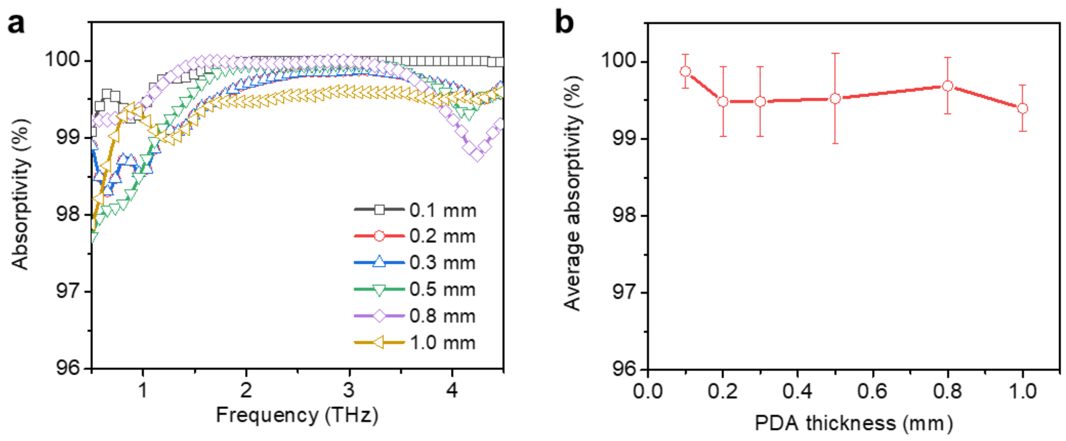

Supplementary Fig. 8 | Absorptivity of the polydodecyl acrylate coated organohydrogel (PDA@OHG) with different polydodecyl acrylate (PDA) thickness in 0.5-4.5 THz band. a Absorptivity curves of the PDA@OHG with different PDA thickness (the thickness of organohydrogel (OHG) layer is 500 μm) in the frequency range of 0.5-4.5 THz. **b** Average absorptivity of PDA@OHG as a function of PDA thickness. Each error bar represents the standard deviation from 5 measurements. Source data are provided as a Source Data file.

The average absorptivity of the PDA@OHG maintains at about 99.7% as the PDA thickness increases from 0.1 mm to 1 mm. Considering the requirement of thin and light for terahertz absorbers, the optimal thickness of the PDA coating is 0.1 mm.

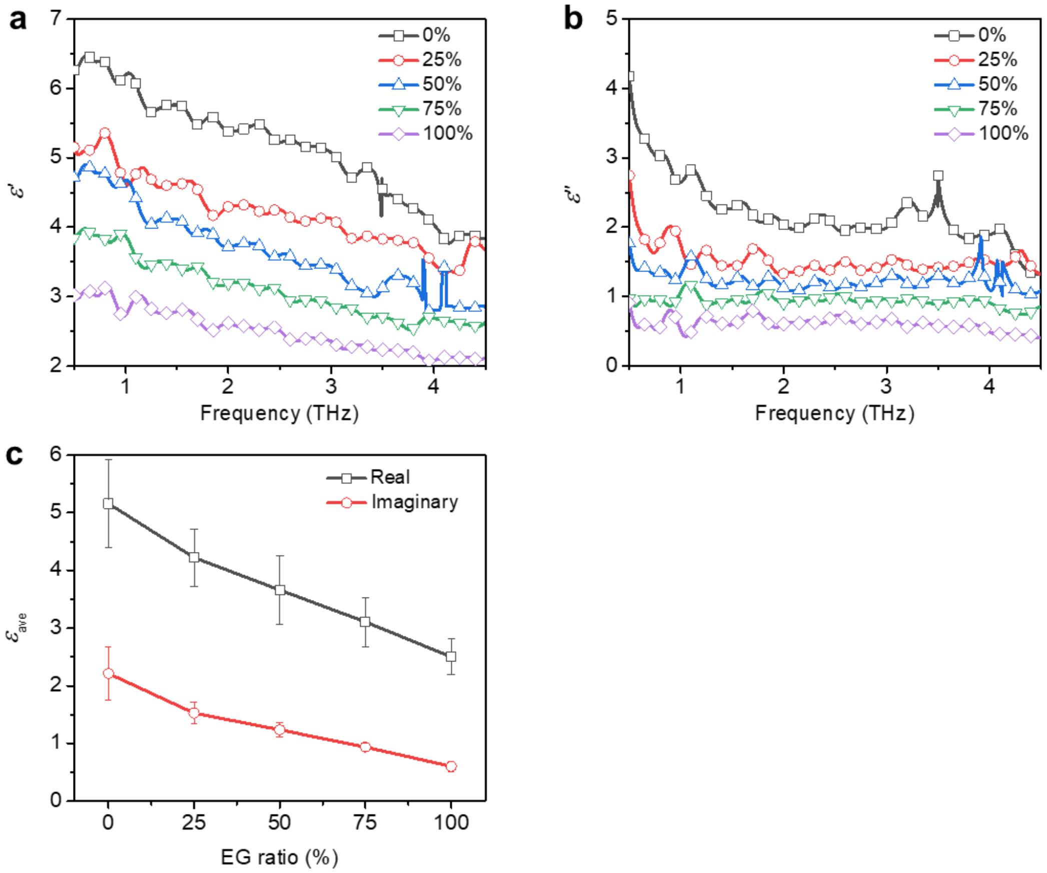

Supplementary Fig. 9 | Complex permittivity of organohydrogel with different ethylene glycol (EG) ratio in 0.5-4.5 THz band. a Real part (ε') of the complex permittivity of the organohydrogel with different EG ratio in 0.5-4.5 THz band. **b** Imaginary part (ε'') of the complex permittivity of the organohydrogel with different EG ratio in 0.5-4.5 THz band. **c** Average permittivity (ε_{ave}) of the organohydrogel as a function of EG ratio in 0.5-4.5 THz band. Each error bar represents the standard deviation from 5 measurements. Source data are provided as a Source Data file.

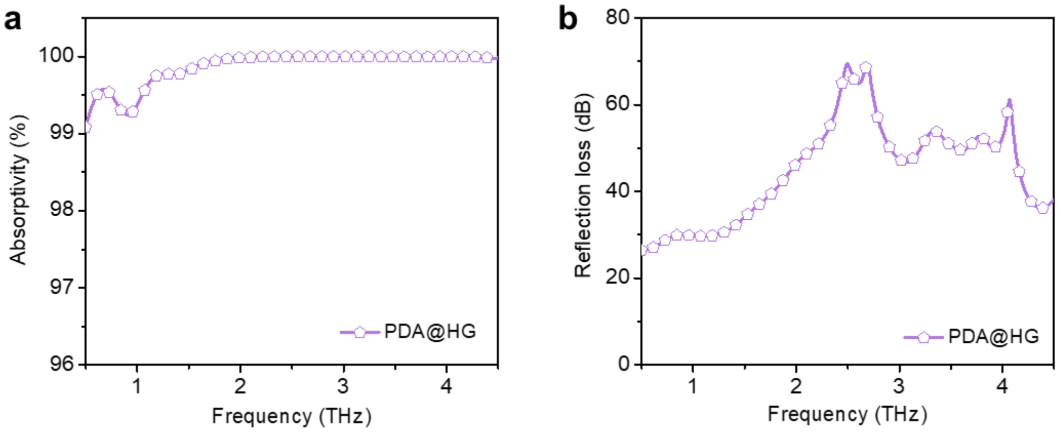

Supplementary Fig. 10 | Terahertz absorption of polydodecyl acrylate coated hydrogel (PDA@HG). a Absorptivity curve of the 700-μm-thick PDA@HG film in 0.5-4.5 THz band. **b** Reflection loss curve of the 700-μm-thick PDA@HG film in 0.5-4.5 THz band. Source data are provided as a Source Data file.

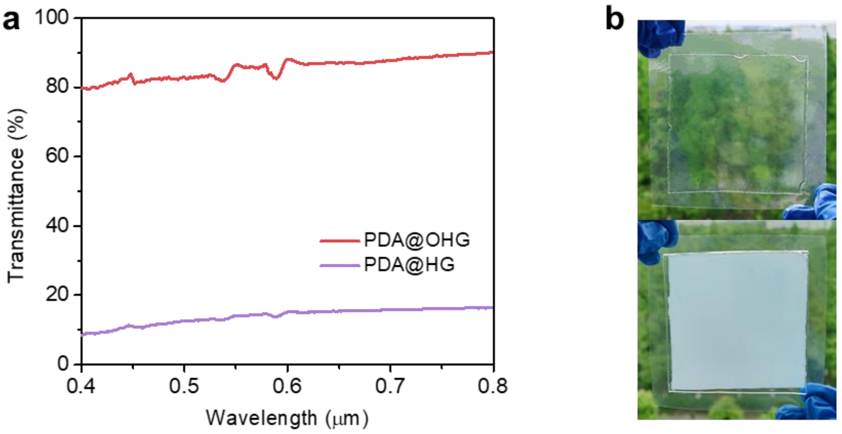

Supplementary Fig. 11 | Visible light transmittance of polydodecyl acrylate coated organohydrogel (PDA@OHG) and polydodecyl acrylate coated hydrogel (PDA@HG). a Transmittance curves of PDA@OHG and PDA@HG with thickness of 500 μm in visible light band (wavelength 0.4-0.8 μm). **b** Photographs of the PDA@OHG (top) and PDA@HG (down). Source data are provided as a Source Data file.

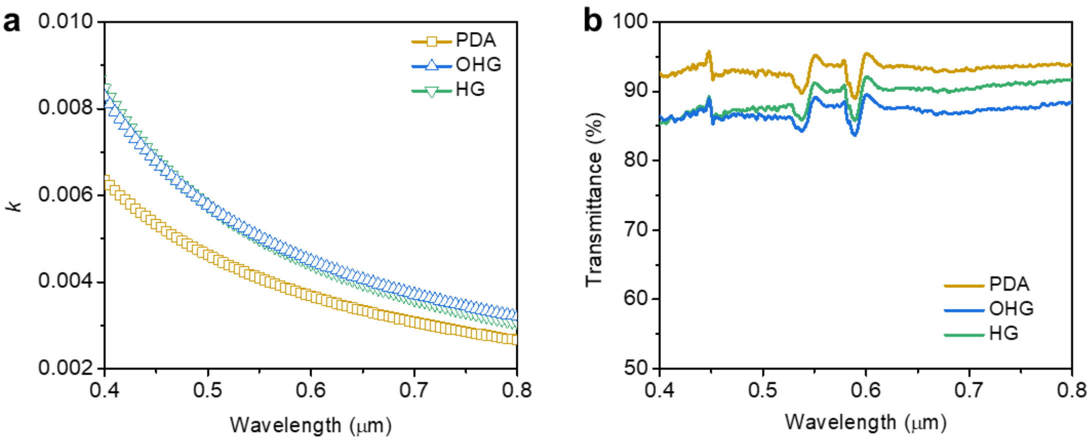

Supplementary Fig. 12 | Visible light transmittance of polydodecyl acrylate (PDA), hydrogel (HG), and organohydrogel (OHG). a Extinction coefficients (*k*) of PDA, HG, and OHG in visible light band (wavelength 0.4-0.8 μm). **b** Transmittance curves of PDA, HG, and OHG in visible light band. Source data are provided as a Source Data file.

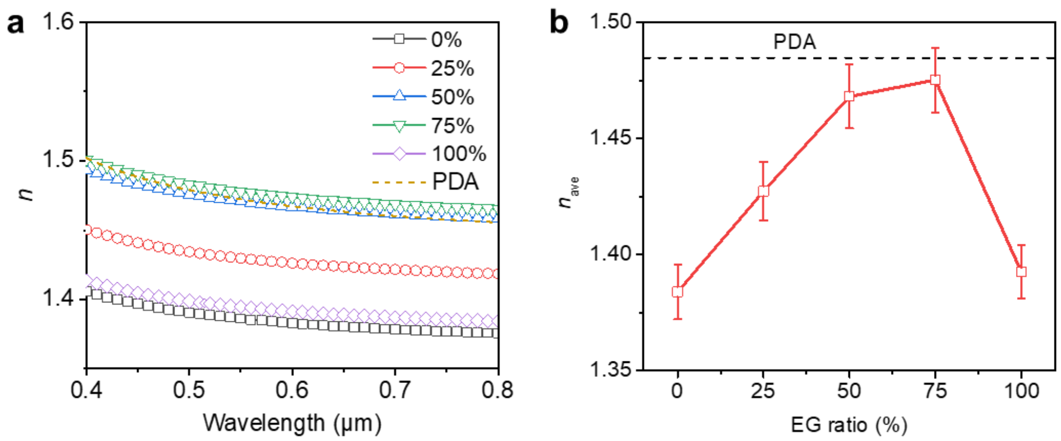

Supplementary Fig. 13 | Visible refractive index of the organohydrogel with different ethylene glycol (EG) ratio. a Refractive index (*n*) of the organohydrogel with different EG ratios in visible light band (wavelength 0.4-0.8 μm). **b** Average refractive index (n_{ave}) of the organohydrogel as a function of EG ratio in visible light band. PDA: polydodecyl acrylate. Each error bar represents the standard deviation from 5 measurements. Source data are provided as a Source Data file.

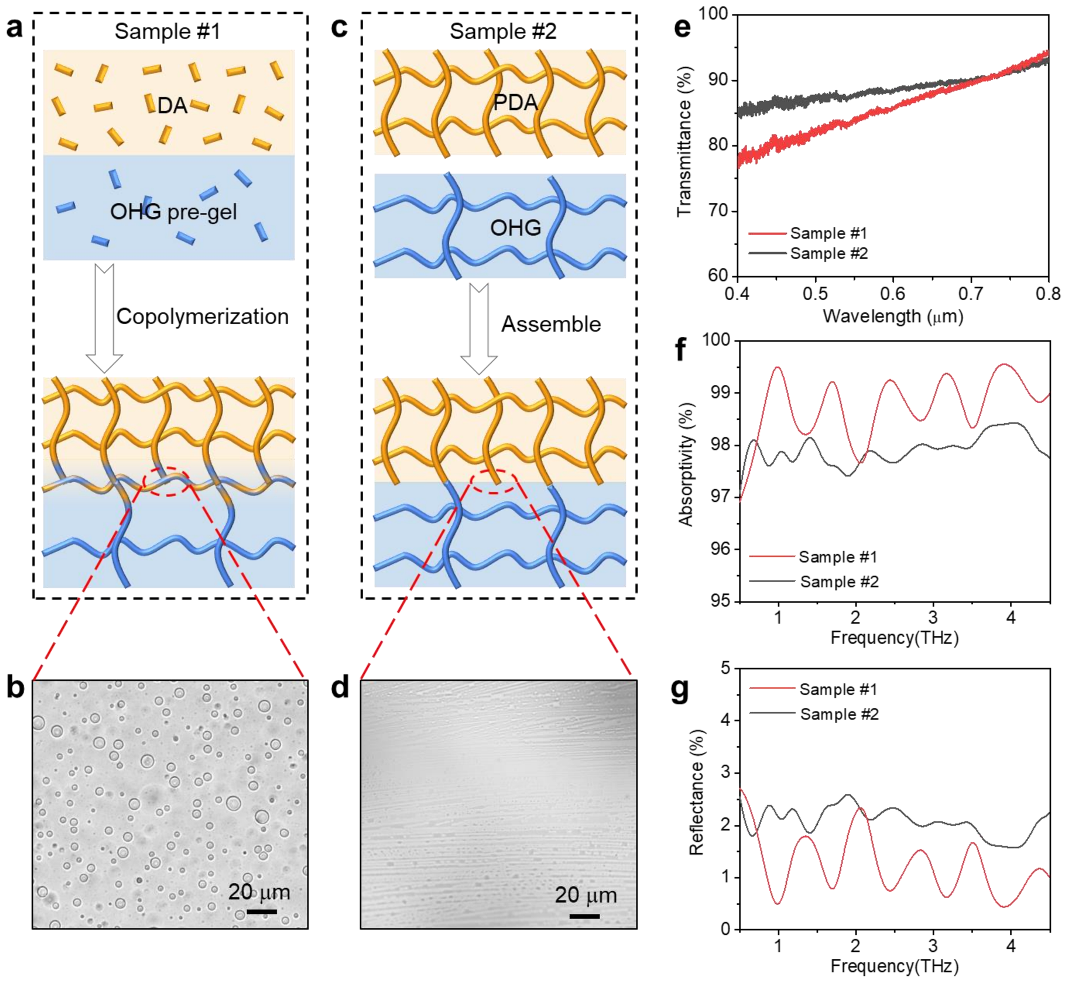

Supplementary Fig. 14 | Comparison of optical properties of the polydodecyl acrylate coated organohydrogel (PDA@OHG) samples with and without microspheres. a, c Preparation schematic of the sample #1 with microspheres (a) and the sample #2 without microspheres (c). **b, d** Optical micrograph of the polydodecyl acrylate (PDA) - organohydrogel (OHG) interface of sample #1 (b) and sample #2 (d). **e** Transmittance of sample #1 and sample #2 in visible light band. **f, g** Absorptivity (f) and reflectance (g) of sample #1 and sample #2 in 0.5-4.5 THz band. Source data are provided as a Source Data file.

In terms of the visible transmittance, the sample #1 with microspheres has a lower value than the sample #2 without microspheres (Supplementary Fig. 14e). This is because the microsphere creates lots of interfaces, increasing the scattering of visible light. In terms of the terahertz absorption, the sample #1 has a higher value than the sample #2 (Supplementary Fig. 14f), which is mainly caused by its lower reflectance (Supplementary Fig. 14g).

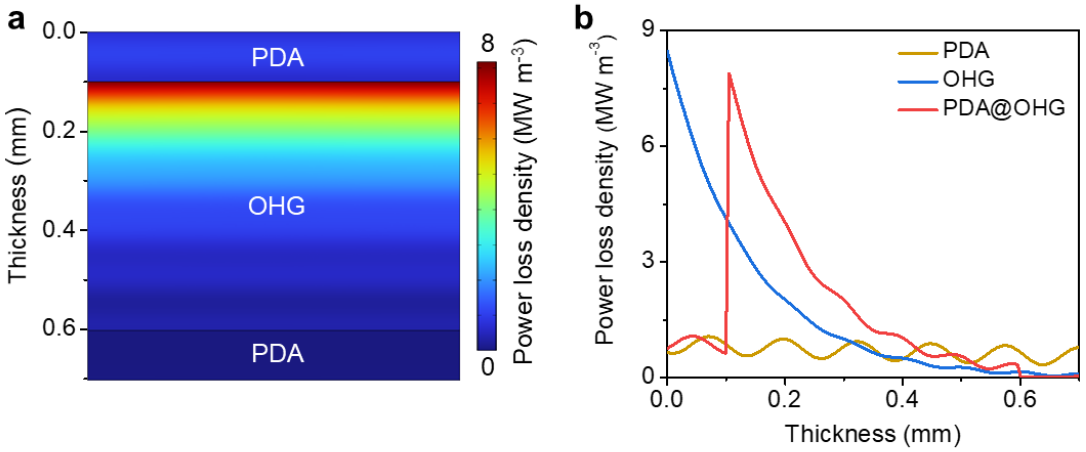

Supplementary Fig. 15 | Finite element simulation of terahertz absorption in polydodecyl acrylate coated organohydrogel (PDA@OHG). a Simulated power loss density profile of PDA@OHG. **b** Variation of power loss density with thickness for polydodecyl acrylate (PDA), organohydrogel (OHG), and polydodecyl acrylate coated organohydrogel (PDA@OHG). Source data are provided as a Source Data file.

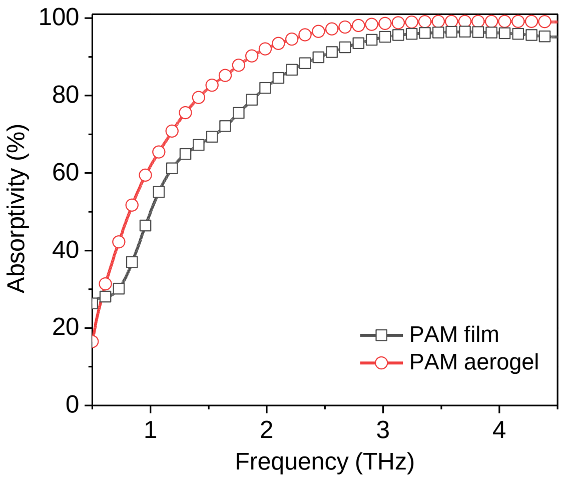

Supplementary Fig. 16 | Absorptivity curves of porous polyacrylamide (PAM) aerogel and dense PAM film at the same weight in 0.5-4.5 THz band. Source data are provided as a Source Data file.

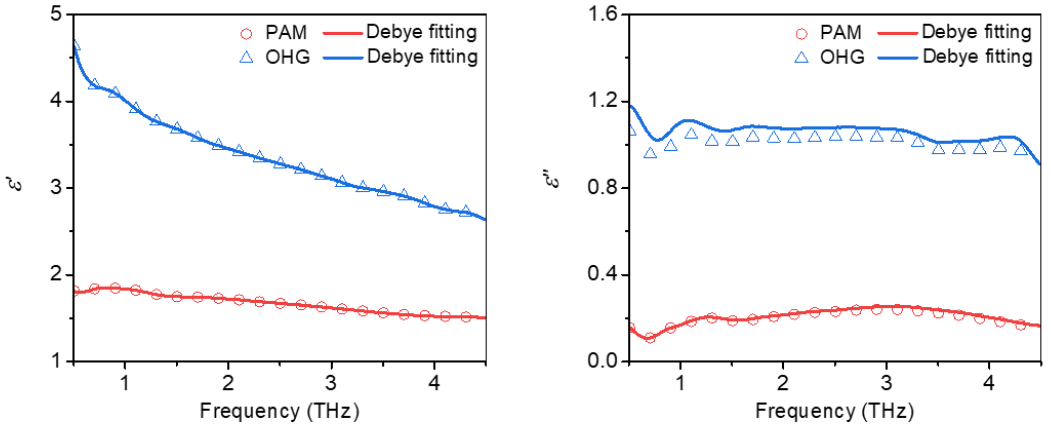

Supplementary Fig. 17 | Debye fitting of the complex permittivity of polyacrylamide (PAM) aerogel and organohydrogel (OHG). a Real part (ε') of the complex permittivity of PAM aerogel and OHG in 0.5-4.5 THz range. **b** Imaginary part (ε'') of the complex permittivity of PAM aerogel and OHG in 0.5-4.5 THz range. The symbols represent the experimental results measured by terahertz (THz) time-domain spectrometer (TDS) system, and the curves represent the fitted results by Debye model. Source data are provided as a Source Data file.

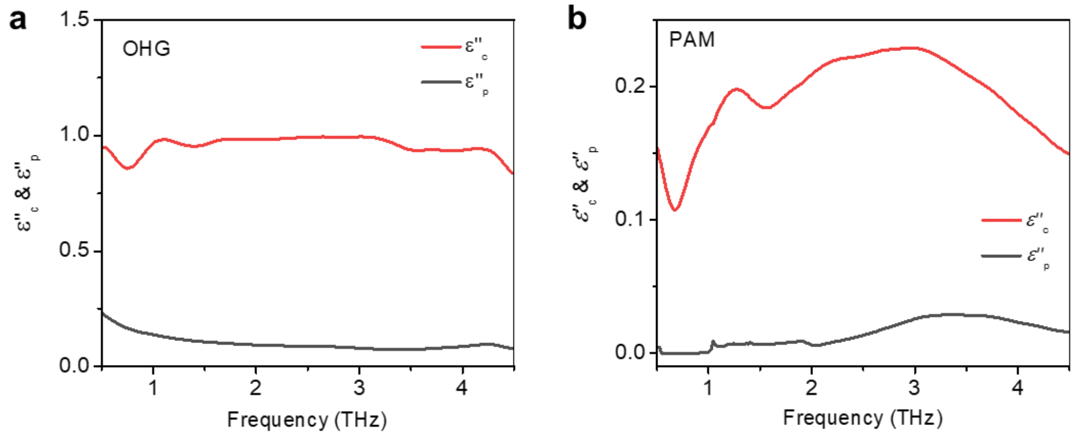

Supplementary Fig. 18 | Conduction/polarization loss of organohydrogel (OHG) and polyacrylamide (PAM) aerogel. a Conduction loss (ε''_c) and polarization loss (ε''_p) curves of the OHG in 0.5-4.5 THz band. **b** Conduction loss (ε''_c) and polarization loss (ε''_p) curves of the PAM aerogel in 0.5-4.5 THz band. Source data are provided as a Source Data file.

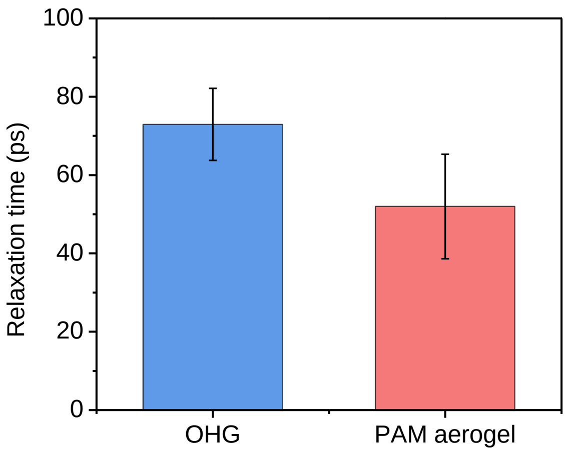

Supplementary Fig. 19 | Relaxation time of organohydrogel (OHG) and polyacrylamide (PAM) aerogel. Each error bar represents the standard deviation from 5 measurements. Source data are provided as a Source Data file.

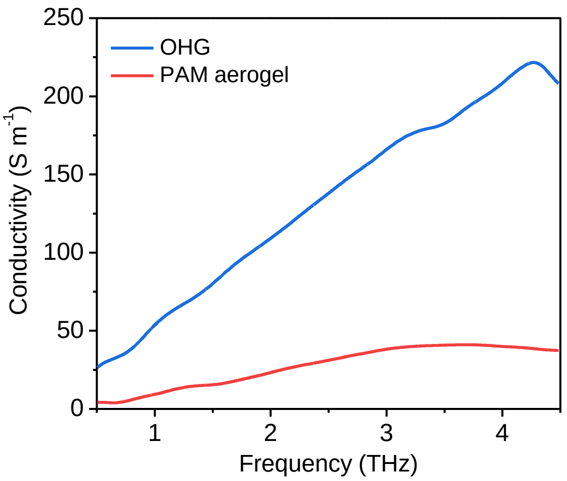

Supplementary Fig. 20 | Conductivity curves of organohydrogel (OHG) and polyacrylamide (PAM) aerogel in 0.5-4.5 THz band. Source data are provided as a Source Data file.

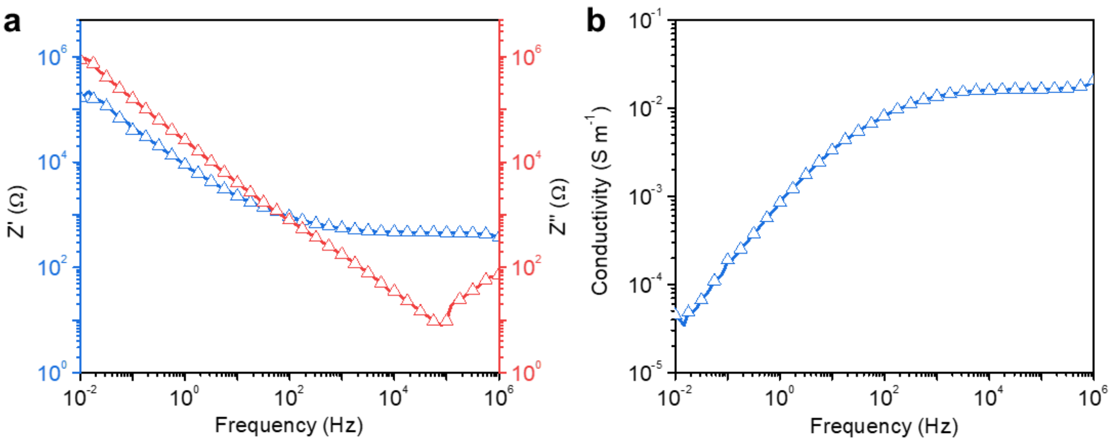

Supplementary Fig. 21 | Conductivity of organohydrogel (OHG) in 10^{-2}-10^{6} Hz band. a Electrochemical impedance spectrum (EIS) of the OHG in 10^{-2}-10^{6} Hz band. **b** Conductivity spectrum of OHG in 10^{-2}-10^{6} Hz band. The conductivity (σ) is obtained by $\sigma = L/(A \times R)$. Where L is thickness, A is area, R is the resistance (real part Z' of the impedance). Source data are provided as a Source Data file.

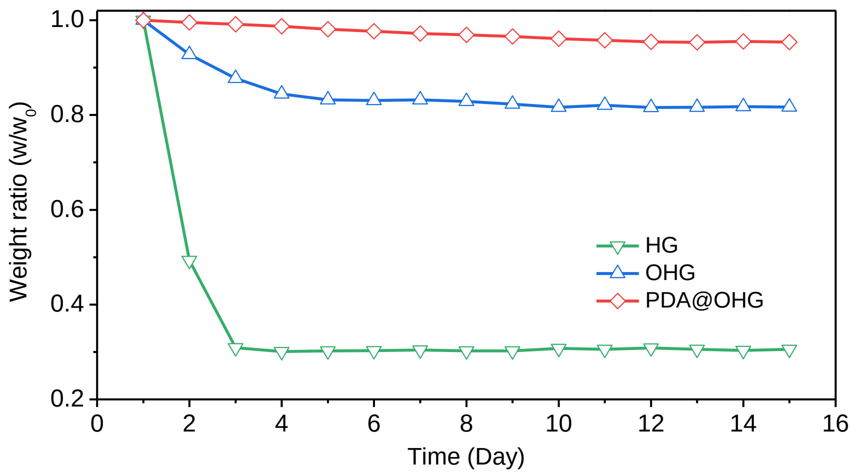

Supplementary Fig. 22 | Changes in weight ratio of hydrogel (HG), organohydrogel (OHG), and polydodecyl acrylate coated organohydrogel (PDA@OHG) with storage time. Storage conditions: temperature 25 °C, humidity 40%). Source data are provided as a Source Data file.

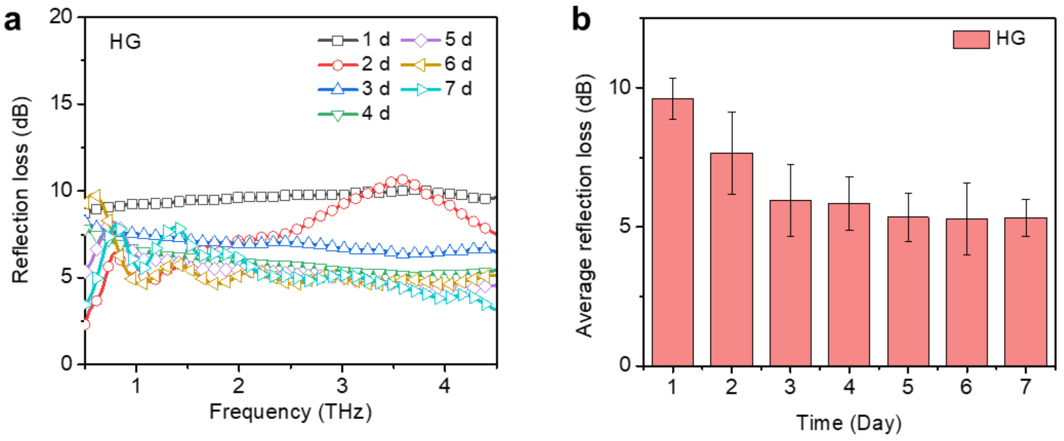

Supplementary Fig. 23 | Change in terahertz absorption of hydrogel (HG) within 7 days. a Reflection loss curves of the HG within 7 days. **b** Change in average reflection loss of the HG with storage time. Each error bar represents the standard deviation from 5 measurements. The sample is stored under environmental conditions (temperature 25 °C, humidity 40%). Source data are provided as a Source Data file.

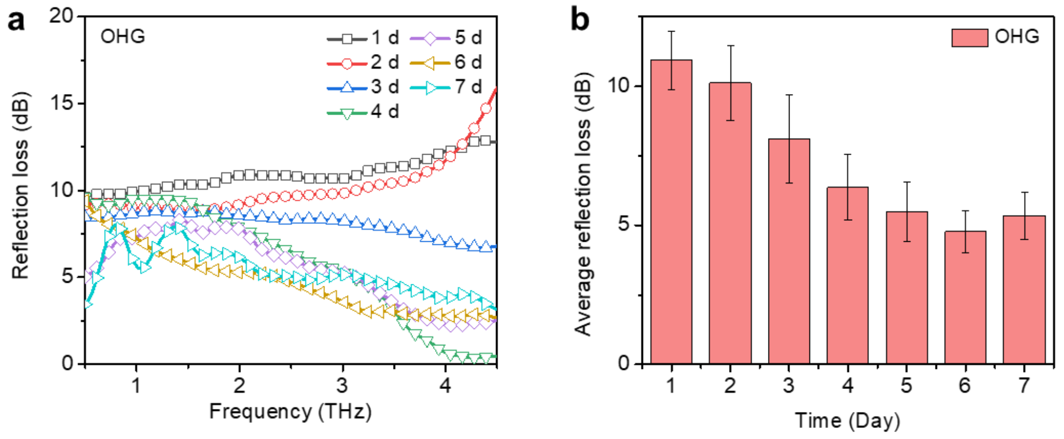

Supplementary Fig. 24 | Change in terahertz absorption of organohydrogel (OHG) within 7 days. a Reflection loss curves of OHG within 7 days. **b** Change in average reflection loss of OHG with storage time. Each error bar represents the standard deviation from 5 measurements. The sample is stored under environmental conditions (temperature 25 °C, humidity 40%). Source data are provided as a Source Data file.

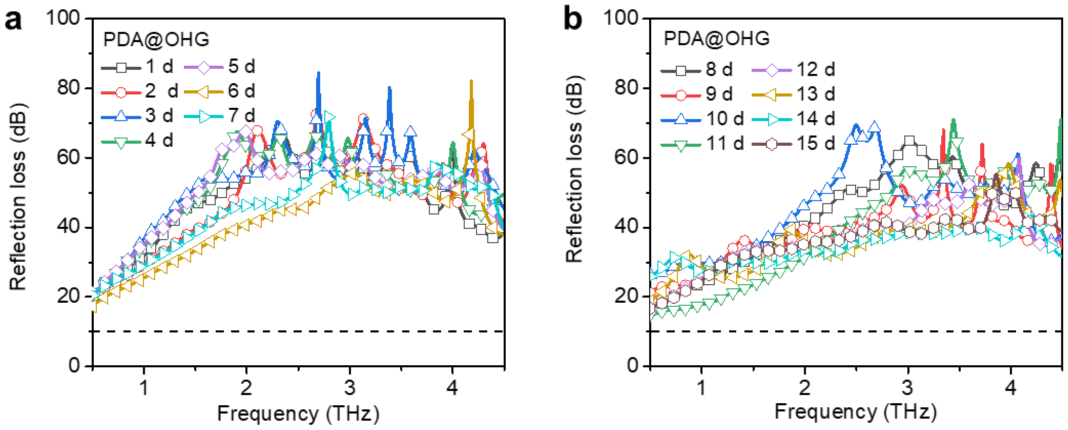

Supplementary Fig. 25 | Reflection loss curves of polydodecyl acrylate coated organohydrogel (PDA@OHG) within 15 days. The sample is stored under environmental conditions (25 °C, humidity 40%). Black dash line represents the commercial standard of effective terahertz absorption. Source data are provided as a Source Data file.

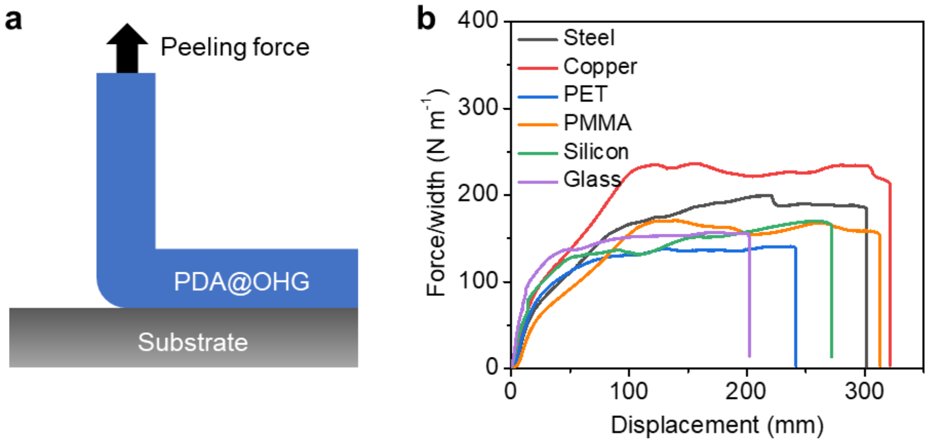

Supplementary Fig. 26 | Peeling test of polydodecyl acrylate coated organohydrogel (PDA@OHG) on diverse substrates. a Schematic of the 90-degree peeling test. **b** Peeling force versus displacement curves of the PDA@OHG on diverse substrates. PET: polyethylene terephthalate, PMMA: polyethylene terephthalate. Source data are provided as a Source Data file.

Supplementary Table 1. Comparison of thickness, frequency band, and average reflection loss between the PDA@OHG in this work and different materials reported in literatures.

**Materials**	**Thickness (mm)**	**Frequency band**	**Average reflection loss (dB)**	**Reference**
Polydodecyl acrylate coated organohydrogel (PDA@OHG)	0.7	0.5-4.5 THz	49.03	This work
Polyacrylic acid (PAA) /$Ti_3C_2T_x$ MXene composite hydrogel	0.13	0.2-2 THz	23.2	[1]
Polyvinyl alcohol (PVA) / reduced graphene oxide (RGO) hydrogel	1	1 THz	2.5	[2]
Carbon-coated SiC/polydimethylsiloxane (PDMS) nanocomposites	0.6	0.5-3 THz	19.0	[3]
Covalently bonded MXene foam	2.5	0.2-1.4 THz	27.3	[4]
Graphene foam	3	0.1-1.6 THz	23	[5]
Carbon nanotube (CNT) / graphene foam	3	0.1-1.6 THz	30	[5]
RGO foam	4	0.1-1.2 THz	19	[6]
MXene / graphene oxide GO foam	4	0.2-2.0 THz	30.6	[7]
Annealed carbonized foam	6	0.25-3.5 THz	47	[8]

Supplementary References

1. Zhu, Y. et al. Multifunctional Ti3C2Tx MXene Composite Hydrogels with Strain Sensitivity toward Absorption-Dominated Electromagnetic-Interference Shielding. *ACS Nano* **15**, 1465-1474 (2021).
2. Xiang, M. et al. Modification of graphene by polypyrrole and ionic liquids for dual-band electromagnetic interference shielding hydrogels. *J. Mater. Sci.* **57**, 10983-10996 (2022).
3. Huang, F. et al. Thermally stable carbon-coated SiC/polydimethylsiloxane nanocomposites for EMI shielding in the terahertz range. *Mater. Res. Bull.* **153** (2022).
4. Fei, Y. et al. Covalent coupling induced-polarization relaxation in MXene-based terahertz absorber for realizing dual band absorption. *Chem. Eng. J.* **461** (2023).
5. Huang, Z. et al. Graphene-Based Composites Combining Both Excellent Terahertz Shielding and Stealth Performance. *Adv. Opt. Mater.* **6** (2018).
6. Huang, Z. et al. Ultra-Broadband Wide-Angle Terahertz Absorption Properties of 3D Graphene Foam. *Adv. Funct. Mater.* **28**, 1704363 (2018).
7. Ma, W. et al. Compressible Highly Stable 3D Porous MXene/GO Foam with a Tunable High-Performance Stealth Property in the Terahertz Band. *ACS Appl. Mater. Interfaces.* **11**, 25369-25377 (2019).
8. Yang, J. et al. Low-cost, flexible and broadband terahertz absorber based on annealed carbonized sponge. *J. Mater. Sci.* **33**, 379-387 (2021).
